# The Impact of COVID-19 Outbreak in Italy on the Sustainable Food Consumption Intention From a “One Health” Perspective

**DOI:** 10.3389/fnut.2021.622122

**Published:** 2021-03-09

**Authors:** Greta Castellini, Mariarosaria Savarese, Guendalina Graffigna

**Affiliations:** ^1^Faculty of Agriculture, Food and Environmental Sciences, Universitá Cattolica del Sacro Cuore, Cremona, Italy; ^2^EngageMinds HUB - Consumer, Food & Health Engagement Research Center, Universitá Cattolica del Sacro Cuore, Milan, Italy

**Keywords:** COVID-19, sustainability, food consumption, food habits, risk perception, food psychology, one health, consumer orientation

## Abstract

Coronavirus disease (COVID-19) is a transmissible illness that was recognized in December 2019 and World Health Organization (WHO) stated a pandemic on 11 March 2020. As no cure has been developed for COVID-19 disease yet, Italy has adopted restrictive measures to avoid the spread of the virus, causing different psychological reactions (e.g., stress, anxiety) that lead people to change lifestyle and in particular the consumer orientation toward food. In addition, the COVID-19 emergency had also affected the Italian economy, causing an 11.3% decrease in GDP (gross domestic product). All these changes gave rise to a sense of instability, but it is known that new possibilities may arise in these situations. In particular, the pandemic could be the turning point to make consumers aware of the close link between human health and the ecosystems, supporting the “One Health” perspective and enhancing the orientation to consumer sustainable food products. However, little is known about how the psychological reactions to COVID-19 emergency have affected the consumers' intention to purchase sustainable food products. In order to answer these questions, a representative sample of 1,004 Italian citizens, extracted by stratified sampling, answered an online survey between May the 12th and 18th 2020. The data were analyzed using ANOVA and contingency tables. The results show that during phase one of COVID-19 disease about 30% of the sample reported that have frequently (often and always) consumed the certified sustainable food products and about 20% of the sample have intention to increase the consumption of them in the next 6 months, percentages that increase among those who feel more vulnerable regarding the risk contagion. Moreover, the psychological impact of the COVID-19 emergency has led to a change in consumers' attitudes, increasing the interest in animal and environmental issues and in human health. These aspects seem to drive the future intention of purchasing sustainable food products. This research highlights how the psychological reactions to the health emergency have changed the consumers' attitudes toward sustainability issues, leading them to follow a more sustainable diet that is recognized as a way to preserve human health, environmental preservation, and animal welfare for present and future generations.

## Introduction

In January 2020, the coronavirus disease 2019 (COVID-19) caused by *severe acute respiratory syndrome coronavirus-2* (SARS-CoV-2) started to spread in Italy and profoundly changed people's habits. As no cure or vaccine has been developed for COVID-19 disease yet, since March 8th the Italian government have adopted restrictive measures to avoid the spread of the virus and the consequent worsening of the health emergency. These measures to prevent and control the spread of COVID-19 disease affected not only the global economic system, causing a drop in the Italian GDP (1), but they also had a profound psychological impact on people, resulting in an increase of stress, depression, anxiety and frustration (2). These psychological reactions to COVID-19 pandemic have changed people's lifestyle and the perception of the Italian economy, defining it as worrying, and at risk of crisis (3). In response to that, the Italian population has greatly changed food consumption and their food purchase patterns (4–6). Indeed, the stress caused by the pandemic and the risk susceptibility levels of contagion led to increase the consumption of comfort food and to raise food intake to feel better, limiting the purchase of fresh vegetables, and fruits (7). In particular, a study carried out during the COVID-19 lockdown on Tunisian consumers (8) has shown that the stress felt during the COVID-19 pandemic and the risk susceptibility levels of contagion have determined a change in the consumers' sustainability attitudes and in particular toward the food wastage. Most people, indeed, declared that nothing of the foods purchased were discarded, setting up a strategy of saving, storing, and eating leftovers. This first study seems to confirm the thinking of some scholars who argued that this pandemic could be a window of opportunity for recognizing the importance of sustainability issues and in particular the close link between human health and the ecosystems (9–12), supporting the “One Health” perspective. In particular, the “One Health” perspective is defined as an approach that identifies the health of people as closely connected to the health of animals and the environmental themes (13). Understanding the importance of these different facets of health could have changed the attitudes and perceptions of Italian consumers toward sustainability issues, leading people to give more importance to sustainable diet (14, 15). Indeed, many studies that dealt with the topic of sustainable food consumption in “One Health” perspective have shown that the importance given to environmental issues, animal welfare, human health and to the individual responsibility in society are the main aspects in influencing the sustainable food consumption and the implementation of sustainable diet (16, 17). Moreover, some studies argued that the change in the consumers' attitudes toward sustainable food products and the consequent implementation of a sustainable diet could be considered as ways to preserve the ecosystem and the human well-being, decreasing the chances of a new pandemic (18, 19). In particular, research conducted in Italy has suggested that to solve the current pandemic and to prevent the future ones is not enough to carry out studies in the field of medicine, immunology and microbiology, but it is necessary to implement a preventive strategy for sustainable development (10, 11) that supports a higher level of clean production in order to reduce the exploitation of the environment and, as result, the factors that cause the spread of COVID-19 disease and other infections in society (20).

Given these premises, it seems clear that researchers and scientific literature have long recognized the importance of interconnection between human health, environmental preservation, and animal welfare, supporting the “One Health” perspective. On the contrary, the Italian consumers seem to have recognized and given importance to these aspects as a reaction to the COVID-19 emergency, leading them to change their orientation toward a more sustainable food consumption. In light of this, it appears necessary to explore changes in consumers' attitudes toward human health, environmental preservation and animal welfare (16, 17), during the COVID-19 crisis. In this way, it is possible to interpret potential future consumption trends and to orient educational strategies devoted to enable better sustainable food consumption (21, 22).

Although there are many studies that have investigated how the emergency situation caused by COVID-19 disease has impacted on food consumption (4, 7, 23), little is known about how the psychological reaction to COVID-19 pandemic affected the current and future intention to change consumption in the direction of choosing food product with a certification of sustainability and more respectful of human health, environmental preservation, animal welfare. In particular, this study focuses on consumer intentions and attitudes as they, as claimed by the Theory of Planned Behavior (24, 25), are the most important predictors of behavior and have been used to forecast a wide range of behaviors including consumer purchase (26, 27). Furthermore, it has been shown that studying intentions and attitudes allows to investigate the actual predisposition that people have toward food consumption. On the contrary, studying food consumption behavior does not allow to understand the real predisposition that the subject has toward a food product or a type of food consumption since it can be influenced by various psychological, situational, and contextual variables that can compromise the realization of an intention (28, 29).

In particular, the aims of this study are: (1) understand how COVID-19 emergency has changed consumer attitudes toward human health, environmental preservation, animal welfare, and the individual responsibility in society (2) understand the association between perceived vulnerability to the contagion risk and the intention to consume certified sustainable food products (3) association between the intention to consume certified sustainable food products and the consumers' attitudes toward human health, environmental preservation, animal welfare, and the individual responsibility in society.

## Materials and Methods

### Participants

Research data were collected via questionnaire that was filled out by a representative sample of the Italian population with sex, age, profession, size of the center, and geographical area extracted by stratified sampling. The percentages relating to the Italian population were taken from the website of ISTAT (30) (https://www.istat.it/it/) and reported in Table 1. The survey was conducted using a CAWI (Computer Assisted Web Interviewing) methodology between May the 12th and 18th 2020. In particular, the data collection period focused on collecting information regarding consumers' orientation toward sustainable food consumption in relation to the evolving health and economic situation of the country. In particular, in the questionnaire there were 2 questions about the self-reported consumption of certified sustainable food products that referred to different phases of COVID-19 in Italy: phase 1, (started in early March), and phase 2, (started in early May). The two questions allow us to portray two different historical-epidemiological periods characterized by distinct attitudes and behavioral reactions, in particular toward sustainable consumption orientations.

**Table 1:** Demographic profiles of the sample (*N* = 1,004).

	***n***	**%**	**% population**
**1. Gender**			
Male	495	49.3	49.3
Female	509	50.7	50.7
**2. Age**			
18–24	101	10.1	10.0
25–34	163	16.3	16.3
35–44	215	21.4	21.5
45–54	228	22.7	22.7
55–59	109	10.8	10.8
60–70	188	18.7	18.8
**3. Education**			
Elementary	3	0.3	–
Junior high	123	12.2	–
Senior high	602	60.0	–
College or university	276	27.5	–
**4. Geographic area**			
North-West	264	23.6	26.3
North-East	187	18.6	18.6
Centre	198	19.7	19.7
South and Islands	355	35.4	35.5
**5. Inhabited center size**			
Until 10,000 inhabitants	314	31.3	32.1
10/100.000 inhabitants	443	44.1	44.0
100/500.000 inhabitants	109	10.9	10.9
More than 500.000	130	12.9	12.9
Missing	8	0.8	
**6. Profession**			
Entrepreneur/freelancer	124	12.4	12.4
Manager/middle manager	38	3.8	3.8
Employee/teacher/military	193	19.2	19.2
Worker/shop assistant/apprentice	211	21.0	21.0
Housewife	151	15.0	15.0
Student	53	5.3	5.3
Retired	79	7.9	7.9
Unoccupied	155	15.4	15.4
**7. Household income level**			
Until 600 €	63	6.2	–
601–900 €	66	6.5	–
901–1,200 €	106	10.5	–
1,201–1,500 €	152	15.1	–
1,501–1,800 €	116	11.6	–
1,801–2,500 €	143	14.3	–
2,501–3,500 €	105	10.4	–
More than 3,501 €	103	10.3	–
Missing	150	15.0	–

The sample is made up of 1,004 subjects of which 495 are male and 509 are female, aged between 18 and 70 years (Mean = 44.45, Standard Deviation = ±13.9). The demographic profile is presented in detail in Table 1. The subjects were randomly selected from the consumers' panel managed by Norstat SRL, a company specialized in collecting data (https://norstat.it/). This study is a part of a broader project (entitled: “Italian citizens' food habits monitoring from a consumer psychology perspective”) aimed at monitoring consumption habits of Italian citizens, to which questions are added based on the most interesting contingent events. This study has been performed in accordance with the Declaration of Helsinki and has been approved by an independent ethics committee of Università Cattolica del Sacro Cuore in Milan (CERPS).

### Measures and Procedure

In particular, in order to answer the research questions, the survey was focused on measuring the following variables (see Supplementary Materials_A):

#### Risk Susceptibility

Participants were asked their perceived risk of being infected by the new COVID-19 virus using a 5-point Likert scale from 1 (very little) to 5 (a lot).

#### Changes of Attitudes After COVID-19

Participants were asked how the emergency situation caused by COVID-19 could possibly have led them to re-evaluate the importance given to environmental issues, animal welfare, human health and responsibility of individuals in society, intended as aspects of “One Health” approach (13). We created 4 *ad hoc* items to assess these aspects starting from the definition of the concept of “One Health.” In particular, the most commonly used definition shared by the US Centers for Disease Control (31) and Prevention and the One Health Commission (32) is: “*One Health is defined as a collaborative, multisectoral, and transdisciplinary approach—working at the local, regional, national, and global levels—with the goal of achieving optimal health outcomes recognizing the interconnection between people, animals, plants, and their shared environment*.” From this definition it is clear that the concept of “One Health” is composed by three specific aspects interconnected with each other: human, animal, and environmental health. To measure these facets, we have created three *ad hoc* items that investigated the importance that people give to environmental issues, animal welfare and human health. Furthermore, the definition talks about “*collaborative approach*” to achieve optimal outcomes referring to the participation and the personal responsibility, paramount aspects to meet the “One Health” principles. In line with this aspect, we have also added a question that measures this issue related to individual responsibility, investigating the importance that people give to the responsibility of individuals in society. These four items were measured using a 5-point Likert scale from 1 (strongly disagree) to 5 (strongly agree).

#### The Orientation Toward the Consumption of Certified Sustainable Food Products

The orientation toward the consumption of certified sustainable food products [identified with the following labels: Sustainable cleaning, Ecocert, Ecolabel, Cruelty free, FSC, Fairtrade, Friends of sea, Dolphin safe, MSC which are, according to the observatory carried out by Nielsen (33), the most used to identify the sustainable food products in Italy] was measured considering the self-reported consumption in the last month (corresponding to phase 1 of COVID-19), starting from phase 2 of COVID-19 and future purchase intentions in the next 6 months. All these items concern the reported sustainable food consumption by people and not data regarding sales. The self-reported consumption in phase 1 of COVID-19 was measured by a 5-point Likert scale from 1 (never purchased) to 5 (always purchased). The self-reported consumption of sustainable food products during phase 2 and the future purchase intentions were assessed surveying whether the perceived buying of these food products had diminished, remained the same or increased. All these questions about the orientation toward food consumption had “I do not know” as an option of answer. The subjects who chose this answer were considered as “missing data” and therefore not reported within the different distributions in order to obtain more readable results.

### Data Analysis

The data analysis is divided into three main parts. In the first section was used a descriptive statistic and in particular frequencies, percentages, means and standard deviations were carried out in order to understand the distribution of different answers relating to the changes of attitudes, after COVID-19, toward the issues of sustainability, the orientation toward the consumption of certified sustainable food products and the different risk susceptibility levels. The second part of analysis focused on assessing the association between the orientation toward the purchase of certified sustainable food products and the different risk susceptibility levels using a series of contingency tables and one-way ANOVA. In particular the one-way ANOVA was used to analyze the effect of the different risk susceptibility levels (independent variable) on the last month's (corresponding to phase 1 of COVID-19 crisis) self-reported purchase of certified sustainable food products (dependent variable) followed by Bonferroni *post-hoc*. Given the categorical nature of the variables and the unbalanced distributions regarding the measure of phase 2 self-reported purchase and the intention to purchase the sustainable food products in the next 6 months we carried out a series of contingency tables inspecting the Pearson's Chi-square and the standardized residuals. The standardized residuals are calculated as the difference between observed and expected counts of a cell divided by an estimate of its standard deviation. Since they are asymptotically normally distributed with a mean of 0 and standard deviation of 1 under the null hypothesis of independence, as a general rule of thumb cells with an absolute value of standard residuals equal or above 2 can be considered to significantly contribute to the general chi-square value (34). The contingency tables were also used in the third and final part of the analysis which is aimed at exploring the association between the future intentions to purchase the sustainable food products and the importance given to environmental issues, animal welfare, human health and the role of individuals in society after COVID-19 emergency. Moreover, we have analyzed the socio-demographic differences between those who have intention to maintain, increase or decrease the consumption of certified sustainable food products in the next 6 months considering the age (18–38 years old; 39–52 years old; >53 years old), gender, income level (low = 600–1,800€; medium = 1,801–3,500; high >3,501), education level (low = elementary and junior high; medium = senior high; high = college or university) and the geographical area of residence (North-West; North-East; Centre; South and Islands). These analyses were carried out using Pearson's χ^2^ to test the hypothesis that the distributions are equal across groups. Whenever the χ^2^ resulted significant, column percentages were confronted as *post-hoc* with a *z-*test (corrected with Bonferroni method), as suggested by Sharpe (35) and were graphed.

To simplify the reading of the variables and to allow comparisons among consumer groups, the continuous variables that measure the risk susceptibility and the change of attitudes toward human health, environmental issues, animal welfare and individual responsibility was divided in bands. In particular, the risk susceptibility was divided into three bands: “low risk susceptibility” in which those who responded that they felt not at risk or at little risk (1 and 2 likert scale points) were grouped together; “medium risk susceptibility” represented by those who answered that they felt neither very nor very at risk (3 likert scale point) and “high risk susceptibility” composed by those who answered that they felt quit or very at risk (4 and 5 likert scale points). As regards questions relating to the attitudes toward environmental issues, animal welfare, human health, and the role of individuals in society the medians of each distribution were calculated and these variables were divided into two bands: “low importance” which includes those who strongly agree, agree or neither agree nor disagree with the proposed statements (1, 2, and 3 likert scale points) and “high importance” which includes those who strongly disagree or disagree with the statements (4 and 5 likert scale points). All analyses have been carried out with IBM SPSS 20.

## Results

The frequency distributions of the items are presented in Table 2. In particular, the results show that most of sample perceives a medium risk susceptibility (42.3%) and only a quarter of the sample has a low risk susceptibility (24.6). Moreover, the results display that this sanitary crisis seems to have shred a light on Italians' awareness toward the sustainability themes. In particular, most of Italians declared that they will increase the importance given to human health (70.4%) followed by the relevance given to environmental issues (63.2%), individual responsibility in society (60%), and animal welfare (55.7%). If we consider the orientation toward the consumption of certified sustainable food products in phase 1 of COVID-19, 30.3% of the Italian population has reported to consume these foods very frequently (often or always). With regard to the orientation toward these products starting from phase 2 and in the next 6 months we note that most people perceived to have kept stable and intend to keep stable the consumption of certified sustainable food products in the next 6 months even if the percentage of those who perceived to have increased or intent to increase this food consumption is greater than those who have perceived to decreased or have intention to decrease them in the next 6 months.

**Table 2:** Frequency distribution of items.

	***n***	**%**	**Mean (SD)**	**Md**	**A**	**K**
**Risk susceptibility (*****N =*** **1,004)**			3.08 (±0.96)	3	−0.19	−0.23
Low *(1–2)*	247	24.6				
Medium *(3)*	424	42.3				
High *(4–5)*	333	33.2				
**Importance of animal welfare (*****N =*** **1,004)**			3.63 (±0.91)	4	−0.36	0.10
Low *(1–2)*	81	8				
Neutral *(3)*	364	36.3				
High *(4–5)*	559	55.7				
**Importance of environmental issues (*****N =*** **1,004)**			3.78 (±0.87)	4	−0.41	0.20
Low *(1–2)*	48	4.8				
Neutral *(3)*	321	32				
High *(4–5)*	635	63.2				
**Importance of human health (*****N =*** **1,004)**			3.91 (±0.80)	4	−0.36	−0.07
Low *(1–2)*	31	3.1				
Neutral *(3)*	267	26.6				
High *(4–5)*	706	70.4				
**Importance of individual responsibility in society (*****N =*** **1,004)**			3.71 (±0.88)	4	−0.40	0.17
Low *(1–2)*	64	6.5				
Neutral *(3)*	337	33.5				
High *(4–5)*	603	60				
Self-Reported **Consumption of certified sustainable food products in the last month (Phase 1 of COVID-19 pandemic) (*****N =*** **930)**			2.69 (±1.31)	3	0.09	−1.17
Never	253	27.3				
Rarely	143	15.3				
Sometimes	252	27.1				
Often	199	21.4				
Always	83	8.9				
Self-Reported **Consumption of certified sustainable food products in phase 2 of COVID-19 pandemic (*****N =*** **770)**						
Decreased	46	5.9				
Remained stable	618	80.2				
Increased	106	13.8				
**Consumption of certified sustainable food products in the next 6 months (*****N =*** **857)**						
Will decrease	36	4.1				
Will remain stable	648	75.6				
Will increase	173	20.2				

*(1) SD, Standard Deviation; Md, median; A, asymmetry; K, kurtosis; (2) the numbers in bracket and written in italics represent the points of the Likert scale that were grouped in order to simplify the reading of the table*.

In order to assess the association between the self-reported frequency of purchase certified sustainable food products (in phase 1 of COVID-19, starting from phase 2 of COVID-19 and in the next 6 months) and the different risk susceptibility levels some contingency tables and one-way ANOVA were carried out. In particular the one-way ANOVA was carried out after checking the normality of distribution. How it is possible to see in Table 2 the kurtosis and asymmetry show a normal distribution. The ANOVA's results showed a main effect of risk susceptibility levels on the consumption of certified sustainable food products in phase 1 of COVID-19 [F_(2, 926)_ = 14.381; *p* < 0.001, η2 = 0.03]. In particular, people who have a higher risk susceptibility level perceived to have purchased, in phase 1 of COVID-19, more certified sustainable food products than those who have a medium or low risk susceptibility level (see Table 3).

**Table 3:** Main results from risk susceptibility levels comparison.

	**Risk susceptibility levels**		
	**Low (*n =* 226)** ***Mean (SD)***	**Medium** **(*n =* 386)** ***Mean (SD)***	**High (*n =* 318)** ***Mean (SD)***
Self-Reported Consumption of certified sustainable food products in the last month (phase 1 of COVID-19 pandemic)	2.42 (±1.29)	2.61 (±1.27)	2.99 (±1.32)
	**Risk susceptibility levels comparison**		
	**Low/medium** ***Diff. mean (SE)***	**Low/high** ***Diff. mean (SE)***	**Medium/high** ***Diff. mean (SE)***
Self-Reported Consumption of certified sustainable food products in the last month (phase 1 of COVID-19 pandemic)	−0.185 (0.108)	−0.572 (0.113)^***^	−0.387 (0.098)^***^

(1) SD, Standard Deviations; (2) Diff mean, differences in means; (3) SE, Standard Errors; (4) Significance in marked with asterisks (*** sig. at p < 0.001).

Considering the orientation toward the consumption of certified sustainable food products in phase 2 of COVID-19 emergency and in the next 6 months, contingency tables show that among those who perceived to have increased in phase 2 the purchase of sustainable food products there is a significantly higher presence of consumers with a high perceived risk of contagion and the same consideration can be used to describe the future intention of consuming certified sustainable food products (see Table 4).

**Table 4:** Results of contingency tables.

**Variables**	**Answers**	**Cell**	**Risk susceptibility levels**			**Row total**
			**Low**	**Medium**	**High**	
Self-Reported Consumption of certified sustainable food products in phase 2 of COVID-19 pandemic *Chi-square = 18.238 (df = 4), p < 0.01*	Diminished	Observed	14	20	12	46
		Expected	10.7	19.1	16.2	
		Std res.	1.0	0.2	−1.0	
	Unchanged	Observed	152	261	204	617
		Expected	143.6	255.9	217.4	
		Std res.	0.7	0.3	−0.9	
	Increased	Observed	13	38	55	106
		Expected	24.7	44.0	37.4	
		Std res.	**−2.4**	−0.9	**2.9**	
		CT	179	319	271	
Self-Reported Consumption of certified sustainable food products in the next 6 months *Chi-square = 24.396 (df = 4), p < 0.001*	Will Diminish	Observed	4	17	14	35
		Expected	8.2	14.9	11.8	
		Std res.	−1.5	0.5	0.6	
	Will Unchanged	Observed	175	278	196	649
		Expected	153.0	276.4	219.6	
		Std res.	1.8	0.1	−1.6	
	Will Increase	Observed	23	70	80	173
		Expected	40.8	73.7	58.5	
		Std res.	**−2.8**	−0.4	**2.8**	
		CT	202	365	290	

*(1) CT, Column Total; Std res, standard residues; df, degrees of freedom; (2) Cells with an absolute value of std. res ≥ 2 are marked in bold*.

**Figure 1:**
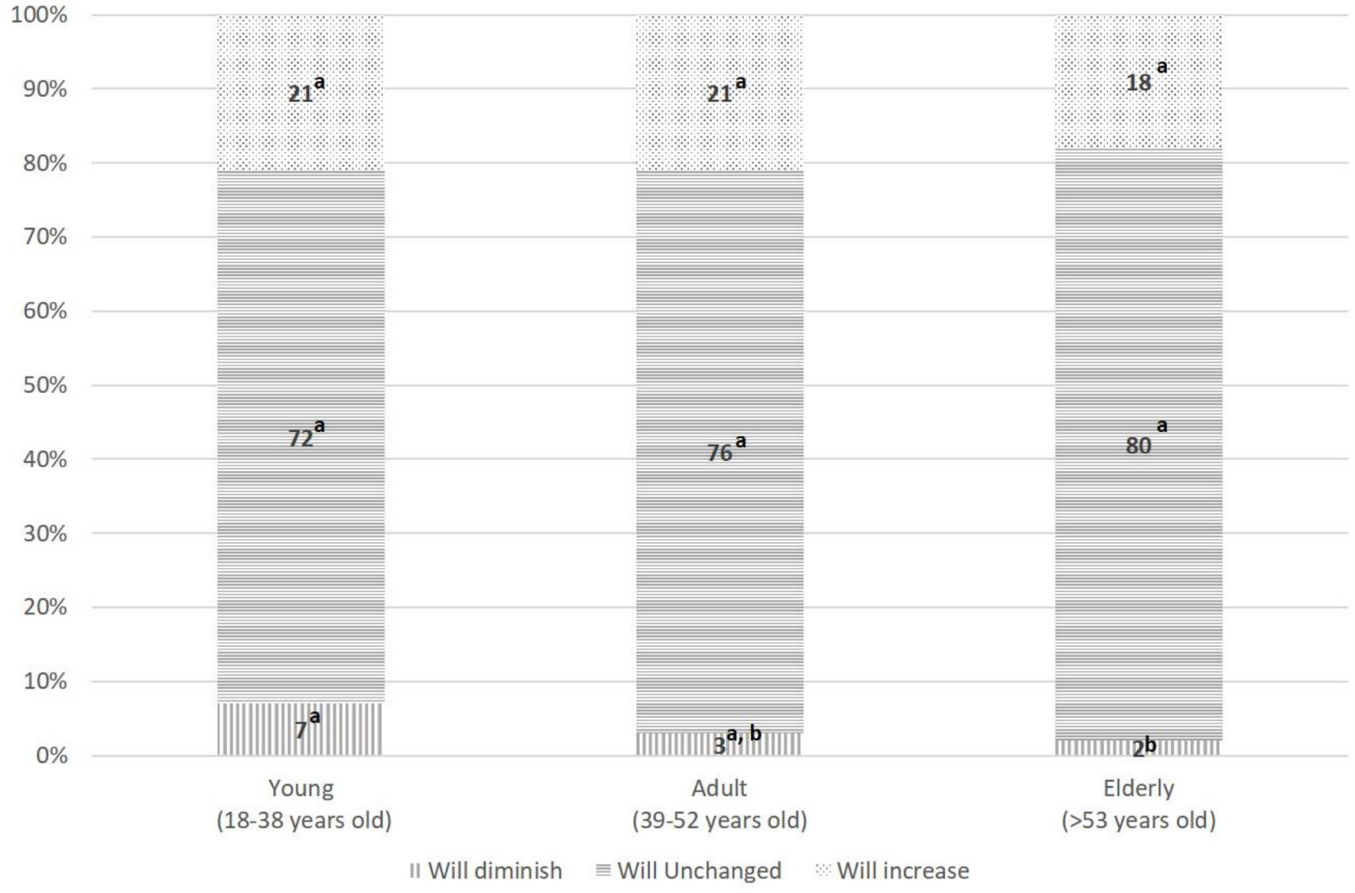
The classification of the consumption of certified sustainable food products in the next 6 months by age. Percentages with the same superscript letter do not differ significantly from each other (*z-*test with Bonferroni correction, *p* > 0.05).

**Figure 2:**
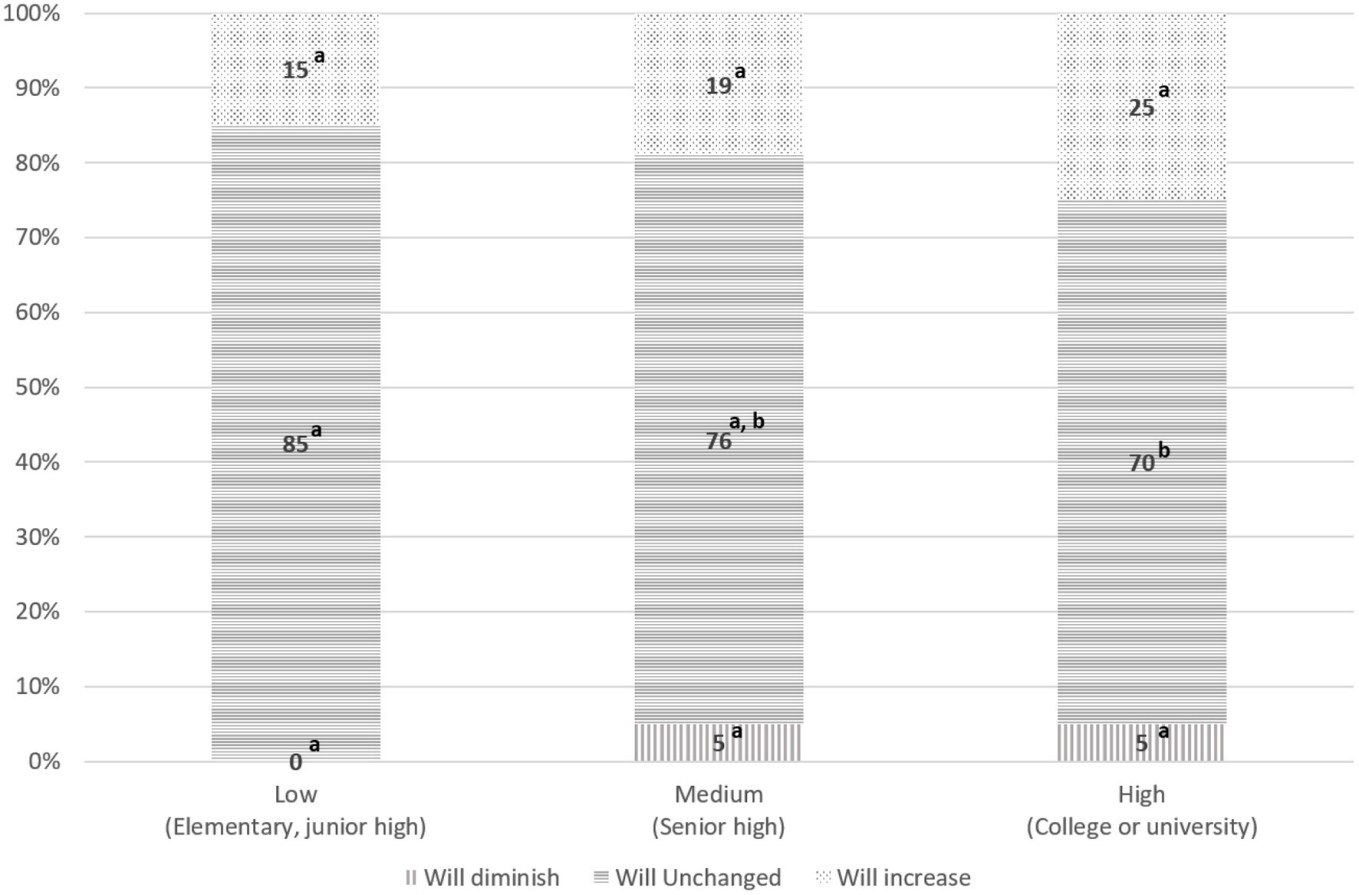
The classification of the consumption of certified sustainable food products in the next 6 months by level of education. Percentages with the same superscript letter do not differ significantly from each other (*z-*test with Bonferroni correction, *p* > 0.05).

Considering the socio-demographic differences of those who intend to increase, not change or decrease the purchase of certified sustainable food products in the next 6 months, we can say that they differ by level of education [*Chi-square* = *11.967 (df* = *4), p* < *0.05)* and by age (*Chi-square* = *10.134(df* = *4), p* < *0.05]*. In particular, among younger subjects there is a higher percentage of people that said that will diminish the consumption of certified sustainable food products in the next 6 months, compared to the oldest individuals (7 vs. 2%, *p* < 0.05; Figure 1). As regards the level of education, we note that those who had a high educational level are less likely to keep stable their sustainable food consumption than people with low level of education (70 vs. 85%, *p* < 0.05; Figure 2) and in particular they are more prone to increase the certified sustainable food consumption in the future. On the contrary, there are no differences for gender (*p* = 0.170), geographical area of residence (*p* = 0.197), and net monthly household income (*p* = 0.073).

Regarding the relationship between changes in attitude toward human health, animal welfare, environmental issues and individual responsibility, after the emergency from COVID-19, and the future orientation toward certified sustainable food, the contingency tables show that among those who intend to increase the consumption of certified sustainable food products in the next 6 months there is a higher presence of consumers who have acquired, during COVID-19 emergency, a greater awareness of the importance about environmental issues, animal welfare and the responsibility of individual in society (see Table 5). With regard to the importance given to human health, it is possible to note that the significance of the chi-square is not so high and also there are not strong significant residual values to comment. This data is difficult to evaluate in terms of significance. However, it is possible to say that among those who intend to increase the consumption of certified sustainable food products in the next 6 months there is a higher presence of consumers who have acquired, during COVID-19 emergency, a greater awareness of the importance of human health even if this presence is not so strongly significant as the other values.

**Table 5:** Results of contingency tables.

**Variables**	**Levels**	**Cell**	**Consumption of certified sustainable food products in the next 6 months**			**Row Total**
			**Will diminish**	**Will unchanged**	**Will increase**	
Importance of animal welfare *Chi-square = 13.181 (df = 2), p < 0.01*	Low *(1–2–3)*	Observed	19	279	51	349
		Expected	14.3	264.2	70.5	
		Std res.	1.3	0.9	**−2.3**	
	High *(4–5)*	Observed	16	369	122	507
		Expected	20.7	383.8	102.5	
		Std res.	−1.0	−0.8	1.9	
		CT	35	648	173	
Importance of environmental issues *Chi-square = 10.814 (df = 2), p < 0.01*	Low *(1–2–3)*	Observed	18	228	44	290
		Expected	11.9	219.5	58.6	
		Std res.	1.8	0.6	**−2.0**	
	High *(4–5)*	Observed	17	420	129	566
		Expected	23.1	428.5	114.4	
		Std res.	−1.3	−0.4	1.4	
		CT	35	648	173	
Importance of human health *Chi-square = 6.486 (df = 2), p < 0.05*	Low *(1–2–3)*	Observed	13	200	38	251
		Expected	10.3	189.8	51.0	
		Std res.	0.9	0.7	−1.8	
	High *(4–5)*	Observed	22	448	136	606
		Expected	24.7	458.2	123.0	
		Std res.	−0.6	−0.5	1.2	
		CT	35	648	174	
Importance of individual responsibility in society *Chi-square = 15.888 (df = 2), p < 0.001*	Low *(1–2–3)*	Observed	22	259	49	330
		Expected	13.8	249.6	66.5	
		Std res.	**2.2**	0.6	**−2.2**	
	High *(4–5)*	Observed	14	390	124	528
		Expected	22.2	399.4	106.5	
		Std res.	−1.7	−0.5	1.7	
		CT	36	649	173	

*(1) CT, Column Total; Std res, standard residues; df, degrees of freedom; (2) Cells with an absolute value of std. res ≥ 2 are marked in bold; (3) the numbers in bracket and written in italics represent the points of the Likert scale that were grouped in order to simplify the reading of the results*.

## Discussion

This study highlighted how the sanitary emergency caused by COVID-19 disease is raising Italians' attention toward the issue of food consumption sustainability, who are re-evaluating the importance of respecting the environmental, animal welfare, human health and who are reappraising the role of individuals' responsibility in society. This changes in Italians' attitudes appear in line with the scientific principles enshrined in the “One Health” perspective. In particular, the COVID-19 emergency had led Italians to attribute an enhanced importance to the promotion of human health, considering this strongly connected with environmental issues and animal welfare and dependent from the level of individuals' social responsibility. Regarding the perception of vulnerability, most of the Italian population perceive a medium risk of contagion of COVID-19 and just a quarter of them stated that they perceive themselves to be at low risk of contagion. Considering the self-reported consumption of certified sustainable food products, this study shows that in phase 1 of COVID-19 almost a third of the Italian population declared to consume these products frequently (often or always) while in phase 2 of COVID-19 and in the next 6 months most of the sample declared to have kept and to will keep them stable even if those who reported that they have increased or want to increase sustainable consumption are much greater than those who reported to have decreased or intend to decrease them. These results are in line with previous studies which showed how the COVID-19 pandemic raised citizens' concerns about the potential consequences of the intensive exploitation of natural resources on the ecosystem and on the well-being of the world population (9–11). This appears to lead people toward the choice of certified sustainable food product (14, 15). A study carried out in China (36) observed that consumers, due to the COVID-19 crisis, increased the consumption of food products considered healthy, natural and environmentally friendly (37, 38). However, these data are controversial. Other studies conducted in Spain (39, 40) showed that the COVID-19 and containment measures determined by lockdown led to less sustainable nutritional habit of consumers with higher values of Global Warming Potential, Blue Water Footprint and Land Use in respect to the average diets of April and March 2019. The variable results of the available literature on the topic may be interpreted as the potential discrepancy between the consumers psychological attitudes and intention toward a sustainable diet (particularly in a period of sanitary crisis) and actual nutritional behaviors. Many studies have explored the intention-behavior relation in the field of food, demonstrating that it is often moderated by several variables such as actual behavioral control, situational context and psychological variables (41–43). In particular, a recent study carried out in China (44) aiming at understanding the gap between intention to buy and actual consumption of sustainable food during COVID-19 crisis, has showed that high prices of green food, unavailability issues, mistrust issues, and limited knowledge moderated the relationship between intentions to consume sustainable food products and the real purchase. In line with this research, could be assumable that the discrepancy between the studies on intentions toward the consumption of sustainable food products and those based on real behavioral data could be explained by the presence of some moderators related to COVID-19 crisis such as the unavailability of sustainable food products given by the scarcity of food resources during the pandemic. Considering the relationship between the orientation toward the consumption of certified sustainable food products in phase 1 of COVID-19 pandemic and the perceived risk of infection, the study shows that those who perceived to have most frequently purchased the sustainable food products have a higher level of risk susceptibility. Moreover, our study shows that among those who perceived to have increased in phase 2 of COVID-19 disease the consumption of sustainable food products there is a significantly higher presence of consumers with a high perceived risk of contagion, underlining how this psychological emotional aspect affects the orientation toward the consumption of certified sustainable food products. the same results are observable if we consider future purchases. This evidence confirms previous studies that have highlighted how the psychological reaction—for example in terms of stress and fear of contagion—determined a change in food consumption intentions (45, 46). For instance, a research on Tunisian consumers (8) has shown that the stress felt during the COVID-19 lockdown has raised citizens attention toward the importance of opting for a better sustainable diet, with a particular focus on reducing food waste. Another longitudinal research carried out by the International Food Information Council (IFIC) (47) on American consumers has shown that, although the perception of importance of achieving an environmental sustainability was stable compared to past years, the COVID-19 pandemic has led American consumers to becoming better aware about the impact of individuals' decisions in achieving the sustainable goals. Furthermore, an Italian study (48) has analyzed how the fear of reduced food availability during the COVID-19 pandemic has led consumers to better preserve and manage purchased food products, by reducing the extent of food waste. Very similar results were achieved in Qatar where the COVID-19 pandemic has not only greatly reduced food waste but has also drastically changed the consumption behavior of people who have purchased more healthier and sustainable products (49).

Focusing on the future orientation toward the consumption of certified sustainable food products, this study underlines how this orientation is also influenced by socio-demographic characteristics such as age and level of education while income, geographical area of residence and gender do not influence this predisposition. In particular, the study showed that young people (18–35 years old), compared to older ones (>53 years old), had a less predisposition to increase the consumption of sustainable food products. This result is controversial in the current literature. Some studies carried out both in Italy (50) and in other foreign countries (51) confirmed that older people seemed to be more prone than the younger ones to purchase sustainable food products. However, other research conducted in Italy and in Serbia, achieved an opposite result, namely that young people were more predisposed toward the sustainable food products than the older ones (52, 53). From this results we may conclude that the socio-demographic characteristics impact on the intention to buy of sustainable food consumption is not clear. Other situational factors may influence this phenomenon, such as the psychological alarm caused by a sanitary crisis. Furthermore, people with a high level of education (college or university) were more likely to change the consumption of sustainable food products in the future by increasing them, than those with a lower education level. This data seems to be strongly consolidated and supported by previous studies which underlined how people with a higher level of education were more orientated toward the consumption of sustainable food products than those with a lower level of instruction (54, 55).

Finally, it is possible to note that the change in attitude toward sustainable issues during the COVID-19 pandemic affects the future intention to consume certified sustainable food products. In particular, the study shows that the consumers attitudes toward the environmental issues, animal welfare and their appraisal of the individual responsibility in the process affect their intentions to consume sustainable food products in the Italian population. These results are in line with other studies that highlight how the consumption of sustainable products is not only influenced by personal ethic values but also by a social component (16, 56). In particular, some studied demonstrated that those who consider their sustainable food choices as a way to contribute to specific sustainable development-related outcomes are more prone to consume sustainable food products (57, 58). Although it is clear that the crisis caused by COVID-19 has increased people's interest in environmental issues, animal welfare and in individual responsibility, which positively influence the future orientation toward the consumption of certified sustainable food products, the role of health is uncertain. Our results clearly show that among those who intend to increase the consumption of certified food products there are more people who have re-evaluated the importance of human health but it is difficult to describe it as a strong significant result. Furthermore, from the results it can be observed that the importance given to human health seems to have a lower association with the consumption of certified sustainable foods than the other variables considered (animals welfare, environmental issues and individual responsibility). The latter result is confirmed by some previous studies (59, 60) that have noted how the egoistic motivations (i.e., health consciousness and food safety) have a significant effect on the intention to purchase products that support sustainable consumption patterns (e.g., consumption of local products, consumption of organic products) but to a lesser extent than altruistic motivations related to respect for the environment and animals. Nonetheless, this evidence suggests that Italians perceive an association between sustainable consumption and the protection of their health even if this awareness should be reinforced.

In conclusion, this study showed that the COVID-19 sanitary emergency has not only increased citizens' awareness toward the issue of sustainability but the sense of vulnerability to the risk of contagion has also led Italians to a greater intention to consume certified sustainable food products during phase 1 and phase 2 of COVID-19 pandemic. This appears also influencing their future consumption intentions. This research, therefore, points out how the crisis caused by COVID-19 can represent an unrepeatable opportunity to educate and involve the consumer toward sustainable diets, exploiting the positive attitudes of consumers toward sustainability issues. For these reasons, this is the moment in which policy makers and food companies should create opportunities for discussion with the consumer, creating workshops or dedicated web platforms. They have the opportunity to take advantage of this “teachable moment” (61), that if properly exploited can push people toward significant change in consumption behavior. In particular, it would be advisable to exploit the openness that older people with a high level of education have toward the consumption of sustainable food products, making them informed citizens who in turn can positively influence the attitudes and orientations of younger people who are less inclined to this type of consumption. Furthermore, these educational moments could be an opportunity to emphasize the concept of “One Health” by highlighting how the safeguarding of our health is closely linked to animal and environmental well-being by promoting sustainable diet as a means of preserving the natural resources and human health (21, 22). Now all the stakeholders of food supply chain have the opportunity -but also the necessity- to rethink on holistic approaches for improving the relationship with the environment that will lead people toward sustainable consumption as a way to preserve the ecosystem and the personal health of present and future generations.

Although this research produced interesting results, it has some limitations. The frequency of consumption of certified sustainable food products is based on self-reported items, which allows the researchers to grasps individual elaboration of this phenomenon and real people's perceived intentions, however these items do not allow to testifying the real consumption behavior. Moreover, the questions regarding the certified food products considered them as a single category that included all sustainable food products without take into account the different types of sustainable labels or product categories. This decision allowed the subjects to have clear examples of certified sustainable food, even if the certifications chosen are not all the possible ones. Moreover, future research could be implemented to deepen these results. In particular, given the controversial intention-behavior relationship, it would be interesting to understand if some psychological (such as social-influence, involvement in food etc.) and situational variables (related to the COVID-19 pandemic) can influence this relationship. It would also be interesting to understand how these variables differ in predicting diverse types of sustainable food products or particular sustainable labels. Finally, despite the presence of many studies that have investigated how COVID-19 has changed consumption habits, the majority of them focused on understanding how negative emotions caused by COVID-19 have worsened food habits, not considering what people have learned from this sanitary emergency. The Coronavirus will not be the last epidemic to hit us therefore it is necessary a preventive strategy based on the key issues that we learned from this pandemic. In this sense, further studies that focus on the “lessons taught” by COVID-19 (as this work) are necessary in order to exploit this knowledge to prevent possible future pandemics.

## Data Availability Statement

The data analyzed in this study is subject to the following licenses/restrictions: The dataset presented in the study is available on request. Requests to access these datasets should be directed to GC, Greta.castellini@unicatt.it.

## Ethics Statement

The studies involving human participants were reviewed and approved by Ethics committee of Università Cattolica del Sacro Cuore in Milan (CERPS). The patients/participants provided their written informed consent to participate in this study.

## Author Contributions

GC: conceptualization, methodology, data curation, formal analysis, and writing—original draft. MS: validation, investigation, and writing—review and editing. GG: writing—review and editing and supervision. All authors have approved the final article.

## Conflict of Interest

The authors declare that the research was conducted in the absence of any commercial or financial relationships that could be construed as a potential conflict of interest.

## References

[1] OECD. Economic Outlook. Available online at: http://www.oecd.org/economic-outlook/june-2020/ (accessed June 30, 2020).

[2] Duan L Zhu G. Psychological interventions for people affected by the COVID-19 epidemic. Lancet Psychiatry. (2020) 7:300–2. 10.1016/S2215-0366(20)30073-0 32085840 PMC7128328

[3] Cerami C Santi GC Galandra C Dodich A Cappa SF Vecchi T. Covid-19 outbreak in Italy: are we ready for the psychosocial and the economic crisis? Baseline findings from the psycovid study. Front Psychiatry. (2020) 11:556. 10.3389/fpsyt.2020.00556 32587539 PMC7297949

[4] Mattioli AV Ballerini Puviani M Nasi M Farinetti A. COVID-19 pandemic: the effects of quarantine on cardiovascular risk. Eur J Clin Nutr. (2020) 74:852–5. 10.1038/s41430-020-0646-z 32371988 PMC7199203

[5] Di Renzo L Gualtieri P Pivari F Soldati L Attinà A Cinelli G. Eating habits and lifestyle changes during COVID-19 lockdown: an Italian survey. J Transl Med. (2020) 18:1–15. 10.1186/s12967-020-02399-5 32513197 PMC7278251

[6] Mattioli AV Sciomer S Cocchi C Maffei S Gallina S. “Quarantine during COVID-19 outbreak: changes in diet and physical activity increase the risk of cardiovascular disease.” *Nutr Metab Cardiovasc Dis*. (2020) 30:1409–17. 10.1016/j.numecd.2020.05.020 32571612 PMC7260516

[7] Di Renzo L Gualtieri P Cinelli G Bigioni G Soldati L Attinà A. Psychological aspects and eating habits during COVID-19 home confinement: results of EHLC-COVID-19 Italian online survey. Nutrients. (2020) 12:2152. 10.3390/nu12072152 32707724 PMC7401000

[8] Jribi S Ben Ismail H Doggui D Debbabi H. COVID-19 virus outbreak lockdown: what impacts on household food wastage? Environ Dev Sustain. (2020) 22:3939–55. 10.1007/s10668-020-00740-y 32837271 PMC7166255

[9] Jones KE Patel NG Levy MA Storeygard A Balk D Gittleman JL. Global trends in emerging infectious diseases. Nature. (2008) 451:990–3. 10.1038/nature06536 18288193 PMC5960580

[10] Coccia M. How (un)sustainable environments are related to the diffusion of COVID-19: the relation between coronavirus disease 2019, air pollution, wind resource and energy. Sustainability. (2020) 12:9709. 10.3390/su12229709

[11] Coccia M. Factors determining the diffusion of COVID-19 and suggested strategy to prevent future accelerated viral infectivity similar to COVID. Sci Total Environ. (2020) 729:138474. 10.1016/j.scitotenv.2020.138474 32498152 PMC7169901

[12] Hakovirta M Denuwara N. How COVID-19 redefines the concept of sustainability. Sustain. (2020) 12:3727. 10.3390/su12093727

[13] World Health Organization (WHO). One Health. (2017). Available online at: https://www.who.int/westernpacific/news/q-a-detail/one-health (accessed October 4, 2020).

[14] Sarkis J Cohen MJ Dewick P Schröder P. A brave new world: lessons from the COVID-19 pandemic for transitioning to sustainable supply and production. Resour Conserv Recycl. (2020) 159:104894. 10.1016/j.resconrec.2020.104894 32313383 PMC7164912

[15] Cohen MJ. Does the COVID-19 outbreak mark the onset of a sustainable consumption transition? Sustain Sci Pract Policy. (2020) 16:1–3. 10.1080/15487733.2020.1740472

[16] Grunert KG Hieke S Wills J. Sustainability labels on food products: consumer motivation, understanding and use. Food Policy. (2014) 44:177–89. 10.1016/j.foodpol.2013.12.001

[17] Antonetti P Maklan S. Feelings that make a difference: how guilt and pride convince consumers of the effectiveness of sustainable consumption choices. J Bus Ethics. (2014) 124:117–34. 10.1007/s10551-013-1841-9

[18] Ventura D de FL di Giulio GM Rached DH. Lessons from the Covid-19 pandemic: sustainability is an indispensable condition of global health security. Ambient Soc. (2020) 23:e0108. 10.1590/1809-4422asoc20200108vu2020l3id

[19] Moriarty P Honnery D. New approaches for ecological and social sustainability in a post-pandemic world. World. (2020) 1:191–204. 10.3390/world1030014

[20] Arora NK Mishra J. COVID-19 and importance of environmental sustainability. Environ Sustain. (2020) 3:117–9. 10.1007/s42398-020-00107-z PMC7220565 38624321

[21] Lawson PJ Flocke SA. Teachable moments for health behavior change: a concept analysis. Patient Educ Couns. (2009) 76:25–30. 10.1016/j.pec.2008.11.002 19110395 PMC2733160

[22] Marks L Ogden J. Evaluation of an online “teachable moment” dietary intervention. Health Educ. (2017) 117:39–52. 10.1108/HE-02-2016-0007

[23] Pellegrini M Ponzo V Rosato R Scumaci E Goitre I Benso A. Changes in weight and nutritional habits in adults with obesity during the “lockdown” period caused by the COVID-19 virus emergency. Nutrients. (2020) 12:2016. 10.3390/nu12072016 32645970 PMC7400808

[24] Ajzen I. From intentions to action: a theory of planned behavior. In Kuhl J Beckman J editors. Action Control: From Cognitions to Behaviors. New York, NY: Springer (1985). p.11–39.

[25] Ajzen I. The theory of planned behavior. Org. Behav. Hum. Decis. Process. (1991) 50, 179–211. 10.1016/0749-5978(91)90020-T

[26] Soon JM Wallace C. Application of theory of planned behaviour in purchasing intention and consumption of Halal food. Nutr Food Sci. (2017) 47:635–47. 10.1108/NFS-03-2017-0059

[27] Fleseriu C Cosma SA Bocănet V. Values and planned behaviour of the Romanian organic food consumer. Sustain. (2020) 12:1722. 10.3390/su12051722

[28] Birch D Memery J. Tourists, local food and the intention-behaviour gap. J Hosp Tour Manag. (2020) 43:53–61. 10.1016/j.jhtm.2020.02.006

[29] Sultan P Tarafder T Pearson D Henryks J. Intention-behaviour gap and perceived behavioural control-behaviour gap in theory of planned behaviour: moderating roles of communication, satisfaction and trust in organic food consumption. Food Qual Prefer. (2020) 81:103838. 10.1016/j.foodqual.2019.103838

[30] ISTAT. Italian National Institute of Statistics. Available online at: www.istat.it (accessed January 1, 2019).

[31] US Centers for Disease Control. One Health Basics. Available online at: https://www.cdc.gov/onehealth/basics/index.html (accessed February 4, 2021).

[32] Prevention and the One Health Commission. Why One Health? Available online at: https://www.onehealthcommission.org/en/why_one_health/what_is_one_health (accessed February 14, 2021).

[33] Nielsen GS1 Italy. Osservatorio Immagino. Le Etichette dei Prodotti Raccontano i Consumi Degli Italiani (2020). Available online at: https://osservatorioimmagino.it/

[34] Haberman SJ. The analysis of residuals in cross-classified tables. Biometrics. (1973) 29:205–20. 10.2307/2529686

[35] Sharpe D. Your chi-square test is statistically significant: now what? Pract Assess Res Eval. (2015) 20:1–10. 10.7275/tbfa-x148

[36] Xie X Huang L Li J Zhu H. Generational differences in perceptions of food health/risk and attitudes toward organic food and game meat: the case of the COVID-19 crisis in China. Int J Environ Res Public Health. (2020) 17:3148. 10.3390/ijerph17093148 32366016 PMC7246561

[37] Pussemier L Larondelle Y Van Peteghem C Huyghebaert A. Chemical safety of conventionally and organically produced foodstuffs: a tentative comparison under Belgian conditions. Food Control. (2006) 17:14–21. 10.1016/j.foodcont.2004.08.003

[38] Murdoch J Marsden T Banks J. Quality, nature, and embeddedness: some theoretical considerations in the context of the food sector. Econ Geogr. (2000) 76:107–25. 10.2307/144549

[39] Batlle-Bayer L Aldaco R Bala A Puig R Laso J Margallo M. Environmental and nutritional impacts of dietary changes in Spain during the COVID-19 lockdown. Sci Total Environ. (2020) 748:141410. 10.1016/j.scitotenv.2020.141410 32798877 PMC7395635

[40] Aldaco R Hoehn D Laso J Margallo M Ruiz-Salmón J Cristobal J. Food waste management during the COVID-19 outbreak: a holistic climate, economic and nutritional approach. Sci Total Environ. (2020) 742:140524. 10.1016/j.scitotenv.2020.140524 32619842 PMC7319639

[41] Terlau W Hirsch D. Sustainable consumption and the attitude-behaviour-gap phenomenon - causes and measurements towards a sustainable development. Int J Food Syst Dyn. (2015) 6:159–74. 10.18461/ijfsd.v6i3.634

[42] Schäufele I Hamm U. Organic wine purchase behaviour in Germany: exploring the attitude-behaviour-gap with data from a household panel. Food Qual Prefer. (2018) 63:1–11. 10.1016/j.foodqual.2017.07.010

[43] Grimmer M Miles MP. With the best of intentions: a large sample test of the intention-behaviour gap in pro-environmental consumer behaviour. Int J Consum Stud. (2017) 41:2–10. 10.1111/ijcs.12290

[44] Qi X Yu H Ploeger A. Exploring influential factors including COVID-19 on green food purchase intentions and the intention–behaviour gap: A qualitative study among consumers in a Chinese context. Int J Environ Res Public Health. (2020) 17:7106. 10.3390/ijerph17197106 32998292 PMC7579444

[45] Castillo CC de A. Analysis of the stress, anxiety and healthy habits in the Spanish COVID-19 confinement. Heal Sci J. (2020) 14:707. 10.36648/1791-809X.14.2.707

[46] Aydemir D Ulusu NN. Influence of the life style parameters including dietary habit, chronic stress and environmental factors and jobs on the human health in relation to COVID-19 pandemic. Disaster Med Public Health Prep. (2020) 14:e36–7. 10.1017/dmp.2020.222 PMC7387745 32580815

[47] International Food Information Council (IFIC). Food & Health Survey. (2020). Available online at: https://foodinsight.org/wp-content/uploads/2020/06/IFIC-Food-and-Health-Survey-2020.pdf (accessed October 1, 2020).

[48] Amicarelli V Bux C. Food waste in Italian households during the Covid-19 pandemic: a self-reporting approach. Food Sec. (2021) 13, 25–37. 10.1007/s12571-020-01121-z 33173548 PMC7644391

[49] Hassen T Ben Bilali H El Allahyari MS. Impact of covid-19 on food behavior and consumption in qatar. Sustain. (2020) 12:6973. 10.3390/su12176973

[50] Coderoni S Perito MA. Sustainable consumption in the circular economy. An analysis of consumers' purchase intentions for waste-to-value food. J Clean Prod. (2020) 252:119870. 10.1016/j.jclepro.2019.119870

[51] Pocol CB Marinescu V Amuza A Cadar RL Rodideal AA. Sustainable vs. unsustainable food consumption behaviour: a study among students from Romania, Bulgaria, and Moldova. Sustain. (2020) 12:4699. 10.3390/su12114699

[52] Annunziata A Agovino M Mariani A. Measuring sustainable food consumption: a case study on organic food. Sustain Prod Consum. (2019) 17:95–107. 10.1016/j.spc.2018.09.007

[53] Kranjac M Vapa-Tankosic J Knezevic M. Profile of organic food consumers. Econ Agric. (2017) 64:497–514. 10.5937/ekoPolj1702497K

[54] Farmery AK Hendrie GA O'Kane G McManus A Green BS. Sociodemographic variation in consumption patterns of sustainable and nutritious seafood in Australia. Front Nutr. (2018) 5:118. 10.3389/fnut.2018.00118 30560133 PMC6287033

[55] Barone B Nogueira RM Guimarães KRLSL de Q Behrens JH. Sustainable diet from the urban Brazilian consumer perspective. Food Res Int. (2019) 124:206–12. 10.1016/j.foodres.2018.05.027 31466642

[56] Han Y Hansen H. Determinants of sustainable food consumption: a meta-analysis using a traditional and a structura equation modelling approach. Int J Psychol Stud. (2012) 4:22. 10.5539/ijps.v4n1p22

[57] Ghvanidze S Velikova N Dodd TH Oldewage-Theron W. Consumers' environmental and ethical consciousness and the use of the related food products information: the role of perceived consumer effectiveness. Appetite. (2016) 107:311–22. 10.1016/j.appet.2016.08.097 27554182

[58] Wang J Nguyen N Bu X. Exploring the roles of green food consumption and social trust in the relationship between perceived consumer effectiveness and psychological wellbeing. Int J Environ Res Public Health. (2020) 17:4676. 10.3390/ijerph17134676 32610596 PMC7369759

[59] Birch D Memery J De Silva Kanakaratne M. The mindful consumer: balancing egoistic and altruistic motivations to purchase local food. J Retail Consum Serv. (2018) 40:21–8. 10.1016/j.jretconser.2017.10.013

[60] Prakash G Choudhary S Kumar A Garza-Reyes JA Khan SAR Panda TK. Do altruistic and egoistic values influence consumers' attitudes and purchase intentions towards eco-friendly packaged products? An empirical investigation. J Retail Consum Serv. (2019) 50:163–9. 10.1016/j.jretconser.2019.05.011

[61] Prochaska JO Di Clemente CC. Transtheoretical therapy: toward a more integrative model of change. Psychotherapy. (1982) 19:276–88. 10.1037/h0088437

