# Supplementary material for: The Impact of COVID-19 Outbreak in Italy on the Sustainable Food Consumption Intention From a “One Health” Perspective
Source: Front Nutr. 2021 Mar 9;8:622122. doi: 10.3389/fnut.2021.622122 (PMC8006295; doi:10.3389/fnut.2021.622122)
Supplement: Supplementary file 1 [file Table_1.docx]

Questionnaire

This questionnaire is aimed at understanding your opinion on how you are experiencing the transition to PHASE 2 of managing the health emergency from COVID-19 and on the effects that this can have on your daily life. In particular, this questionnaire aims to collect information about the sustainable food consumption during the different phases of COVID-19 emergency. We ask you to answer all questions in the questionnaire. there are no "right" or "wrong" answers: what matters is that each answer represents your true opinion regarding this health emergency.

1. ***How much do you feel at risk of being infected by the new Coronavirus? (1= Not at all 5 = A lot at risk).***

| ***Not at all*** | ***A little*** | ***Nor little nor much*** | ***Quite at risk*** | ***A lot at risk*** |
| --- | --- | --- | --- | --- |
| ***1*** | ***2*** | ***3*** | ***4*** | ***5*** |

1. **In the last month, how often have you PURCHASED the certified sustainable food products (Sustainable cleaning, Ecocert, Ecolabel, Cruelty free, FSC, Fairtrade, Friends of sea, Dolphin safe, MSC)?**

Please indicate a value from 1 (never) to 5 (always). If you are unable to define the purchase frequency, select "I don't know".

| ***Never*** |  |  |  | ***Always*** | ***I don’t know*** |
| --- | --- | --- | --- | --- | --- |
| ***1*** | ***2*** | ***3*** | ***4*** | ***5*** | ***6*** |

1. **Starting from the beginning of phase 2 (May 4th, 2020), have you increased or decreased the purchase of the certified sustainable food products (Sustainable cleaning, Ecocert, Ecolabel, Cruelty free, FSC, Fairtrade, Friends of sea, Dolphin safe, MSC)?**

If you don't usually buy it, you can choose the "I don't usually buy it" option

- Decreased
- remained stable
- increased
- I don't usually buy it

1. **Thinking about the next 6 months, in your family you intend to increase, decrease or keep stable the purchase of the certified sustainable food products (Sustainable cleaning, Ecocert, Ecolabel, Cruelty free, FSC, Fairtrade, Friends of sea, Dolphin safe, MSC)?**

- Will Decrease
- Will remain stable
- Will increase
- I don't know

1. **To conclude, thinking ahead, it is possible that people will implement changes in the way they live and see things after experiencing this health emergency from COVID-19**

For each statement reported here indicate its degree of agreement using a scale from 1 (strongly disagree) to 5 (strongly agree)

***In the future you feel that ...***

|  | ***Strongly disagree (1)*** | ***Disagree (2)*** | ***Neither agree nor disagree (3)*** | ***Agree***  ***(4)*** | ***Strongly agree***  ***(5)*** |
| --- | --- | --- | --- | --- | --- |
| I will be more aware of the importance of individual health |  |  |  |  |  |
| I will be more aware of the role of individuals in society |  |  |  |  |  |
| I will give more importance to animal welfare |  |  |  |  |  |
| I will be more aware of environmental issues |  |  |  |  |  |

**Gender**

- Male
- Female

**Age**

|  |
| --- |

**Region of residence**

- Abruzzo
- Basilicata
- Calabria
- Campania
- Emilia Romagna
- Friuli Venezia Giulia
- Lazio
- Liguria
- Lombardia
- Marche
- Molise
- Piemonte
- Puglia
- Sardegna
- Sicilia
- Toscana
- Trentino Alto Adige
- Umbria
- Valle d'Aosta
- Veneto

**Inhabited centre size**

- Up to 10000 inhabitants
- 10001/100000 inhabitants
- 100001/500.000 inhabitants
- More than 500.000 inhabitants
- I don’t know

**Employment**

- Entrepreneur / freelancer
- Manager / official / middle manager
- Employee / teacher / military
- Worker / shop assistant / apprentice
- Housewife
- Student
- Retired
- Unoccupied
- Other

**Level of education**

- Elementary
- Junior high
- Senior high
- College or university

**Can you tell me which of these classes your family's net monthly income falls into?**

- Up to 600 euro
- 601-900 euro
- 901-1200 euro
- 1201-1500 euro
- 1501-1800 euro
- 1801-2500 euro
- 2501-3500 euro
- 3501-4500 euro
- More than 4500 euro
- I prefer not to answer
